# Extinction of Zika Virus and Usutu Virus by Lethal Mutagenesis Reveals Different Patterns of Sensitivity to Three Mutagenic Drugs

**DOI:** 10.1128/AAC.00380-18

**Published:** 2018-08-27

**Authors:** Maria Rosaria Bassi, Raquel Navarro Sempere, Prashansa Meyn, Charlotta Polacek, Armando Arias

**Affiliations:** a Technical University of Denmark, National Veterinary Institute (DTU Vet), Kemitorvet, Lyngby, Denmark; b Statens Serum Institut, Copenhagen, Denmark

**Keywords:** 5-fluorouracil, Usutu virus, Zika virus, error threshold, favipiravir, flavivirus, lethal mutagenesis, mutation frequency, ribavirin

## Abstract

Flaviviruses constitute an increasing source of public health concern, with growing numbers of pathogens causing disease and geographic spread to temperate climates. Despite a large body of evidence supporting mutagenesis as a conceivable antiviral strategy, there are currently no data on the sensitivity to increased mutagenesis for Zika virus (ZIKV) and Usutu virus (USUV), two emerging flaviviral threats. In this study, we demonstrate that both viruses are sensitive to three ribonucleosides, favipiravir, ribavirin, and 5-fluorouracil, that have shown mutagenic activity against other RNA viruses while remaining unaffected by a mutagenic deoxyribonucleoside. Serial cell culture passages of ZIKV in the presence of these compounds resulted in the rapid extinction of infectivity, suggesting elevated sensitivity to mutagenesis. USUV extinction was achieved when a 10-fold dilution was applied between every passage, but not in experiments involving undiluted virus, indicating an overall lower susceptibility than ZIKV. Although the two viruses are inhibited by the same three drugs, ZIKV is relatively more susceptive to serial passage in the presence of purine analogues (favipiravir and ribavirin), while USUV replication is suppressed more efficiently by 5-fluorouracil. These differences in sensitivity typically correlate with the increases in the mutation frequencies observed in each nucleoside treatment. These results are relevant to the development of efficient therapies based on lethal mutagenesis and support the rational selection of different mutagenic nucleosides for each pathogen. We will discuss the implications of these results to the fidelity of flavivirus replication and the design of antiviral therapies based on lethal mutagenesis.

## Abstract (precis)

Flaviviruses constitute an increasing source of public health concern, with growing numbers of pathogens causing disease and geographic spread to temperate climates. Despite a large body of evidence supporting mutagenesis as a conceivable antiviral strategy, there are currently no data on the sensitivity to increased mutagenesis for Zika virus (ZIKV) and Usutu virus (USUV), two emerging flaviviral threats.

## INTRODUCTION

Human disease caused by flaviviruses represents a growing source of global health concern, with elevated numbers of deaths and cases of severe disease (1, 2). The incidence of flavivirus-related disease has increased during recent years. This is possibly related to multiple environmental and socioeconomic factors, such as long-distance spread of pathogenic flaviviruses (e.g., introduction to a different continent) and broader dissemination in temperate climate regions (1, 3, 4). Despite having had limited relevance to public health prior to 2007 (only 14 cases reported), recent large epidemics of Zika virus (ZIKV) in Asia and the Americas have had a major socioeconomic impact. It is estimated that there have been over 1 million cases of infection, leading to several thousand people suffering from severe disease (2, 5). In addition to severe neurological conditions, such as Guillain-Barré syndrome and congenital microcephaly, a wide range of disorders linked to the establishment of persistent infection in different tissues have been documented (6–10).

Without attracting the same level of attention as ZIKV, other emerging flaviviruses are affecting an increasing number of people. In particular, viruses infecting birds, such as West Nile virus (WNV) and Usutu virus (USUV), have been related to recent cases of neurologic disease in temperate countries (11–14). Increased incidence of flaviviral disease seems to be connected to climate change and its impact on the migratory dynamics of birds and the geographic spread of mosquito vectors (15–18). USUV has caused recent epidemics in birds across Europe, with elevated mortality in some species, such as blackbirds, owls, and other wild and captive animals (15, 19). Recent sporadic cases of human disease geographically connected to these outbreaks are raising concerns of USUV becoming a potential threat to global health (11, 13, 15, 18, 20–24).

Lethal mutagenesis has long been proposed as a broad-spectrum strategy to control viral infections, with recent data supporting its feasibility and efficacy *in vivo* (25, 26). The rationale for antiviral therapies based on mutagenesis stems from theoretical studies by Eigen and colleagues (27, 28). These investigations led to the proposal that the elevated error frequencies during RNA virus replication are in the proximity of a maximum tolerated value for viability, namely, the error threshold (28–30). Mutation rates beyond this value would be incompatible with maintaining meaningful genetic information, and thus virus propagation, leading to the extinction of the population in a process known as error catastrophe (28–30). The theory was empirically proven using mutagenic agents that induce increased mutation frequencies in viruses (31–34). A vast repertoire of molecules that exert broad-spectrum antiviral activities linked to viral mutagenesis have been identified, including nucleoside and nonnucleoside compounds (25, 32, 35–40). Mutagenic nucleosides can be incorporated into newly synthesized viral RNA genomes after their intracellular conversion into phosphorylated nucleoside analogues (32, 41–46).

Some of these compounds are currently used at the clinical level; e.g., ribavirin has been extensively used for the treatment of hepatitis C virus (HCV) infection, and favipiravir (also known as T-705 and commercialized as Avigan), has been trialed against influenza virus and Ebola virus disease (45, 47–49). Several recent studies have indicated an association between mutagenesis and antiviral activity *in vivo*. We have demonstrated that the ribonucleoside favipiravir can cure persistent murine norovirus infection in the mouse intestine. This antiviral activity is accompanied by increased mutation frequency and decreased specific infectivity, both signatures of error catastrophe, in samples isolated preceding complete viral clearance (25). Additional evidence of antiviral mutagenesis *in vivo* has been obtained from the analysis of HCV-infected patients treated with ribavirin (50). Larger mutation frequencies accompanied by decreased specific infectivity were also observed in Hantaan virus recovered from infected mice treated with ribavirin (26). Several other studies have provided additional indirect proof of antiviral mutagenesis *in vivo*, further stimulating the development of therapies based on this strategy (51–58).

Several nucleoside analogues have demonstrated antiviral activities against a broad number of flaviviruses, which include ZIKV and WNV (59–65). In particular, ribavirin and favipiravir efficiently inhibit ZIKV infection in different cell culture systems, including human neuronal progenitor cells (59). A correlation between the antiviral activity elicited by these molecules and larger mutation frequencies has been observed for some flaviviruses (62, 63, 65, 66). However, the possible mutagenic activity of these molecules on ZIKV and USUV has not been yet investigated. It also remains unclear whether two different although related pathogens can have different responses to the treatment with the same mutagenic compounds or whether they show distinct sensitivity to them. This information could be relevant in the design of broad-spectrum antiviral therapies against the flaviviruses based on lethal mutagenesis.

Here, we examine the antiviral activities displayed by three nucleoside analogues, all licensed for human use, in cell culture infection with ZIKV and USUV. We observe that ribavirin, favipiravir, and 5-fluorouracil are all inhibitors of both ZIKV and USUV, and that consecutive passage of virus in the presence of these drugs can lead to the complete extinction of infectivity. Notably, the efficacies of these drugs vary depending on the virus. The molecules exhibiting better antiviral efficacies are typically associated with higher mutagenicity of the corresponding virus. However, the relative increases in mutation frequency observed for each drug treatment differ in USUV and ZIKV. We observed the highest mutation frequencies in ZIKV when treated with ribavirin and favipiravir, and in USUV when treated with 5-fluorouracil. The relevance of these results to a better understanding of flavivirus replication, genetic diversity, and the development of prospective antiviral therapies will be discussed.

## RESULTS

### Zika virus replication is suppressed by different ribonucleoside analogues.

**FIG 1:**
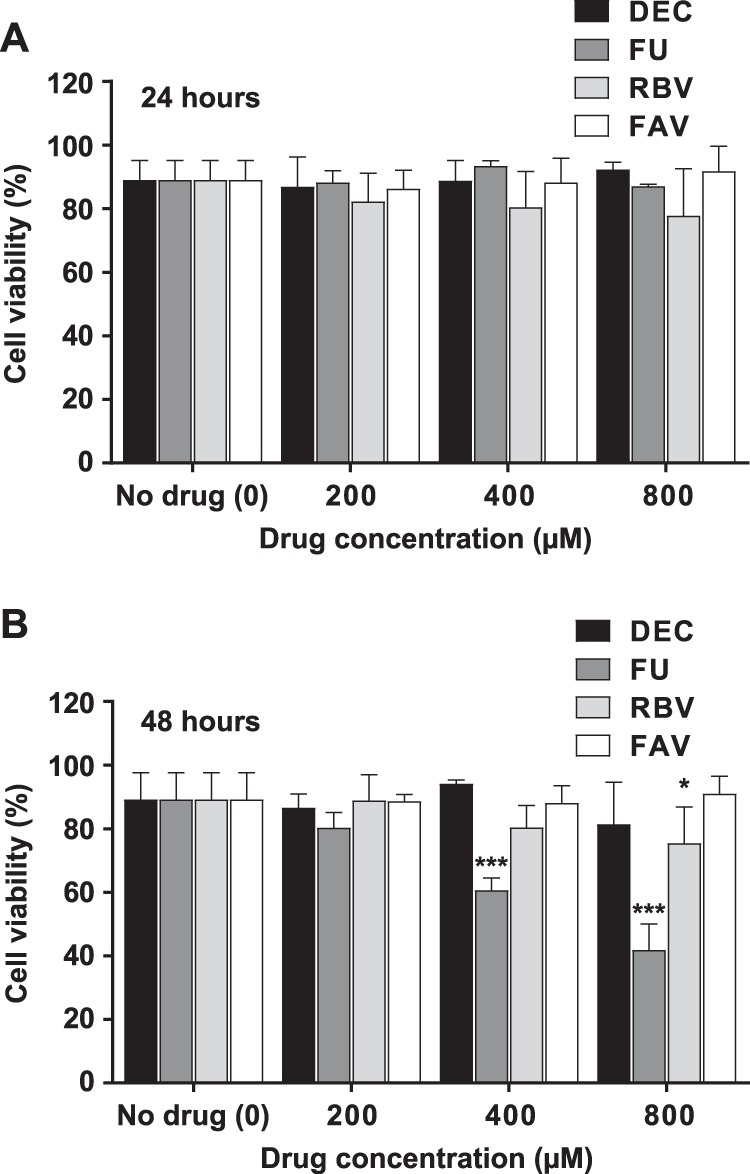
Cell toxicity after treatment with nucleoside drug analogues. The toxicities of decitabine, 5-fluorouracil, favipiravir, and ribavirin upon Vero cells were scored using trypan blue under the microscope. Different concentrations of each drug (200 μM, 400 μM, and 800 μM) were applied to individual cell monolayers. Cell viability values for untreated cell cultures are included in the analysis (represented as 0 μM drug concentration). Cell viability was quantified by determining the proportion of live cells (white) relative to the total (white and blue) in each well. The percentages of live cells after 24 h (A) and 48 h (B) of exposure to each drug at the concentrations indicated are represented. Statistical significant differences in viability rates found in treated cells relative to untreated cell cultures are indicated (*, *P* < 0.05; ***, *P* < 0.001; 2-way ANOVA).

To investigate whether ZIKV replication could be affected by increased mutagenesis, we tested four different compounds known to be mutagenic for diverse viruses, 5-fluorouracil, ribavirin, favipiravir, and decitabine (25, 32, 35, 37–39). Decitabine is an inhibitor of human immunodeficiency virus (39, 67). Its antiviral activity has been associated with lethal mutagenesis, and it is possibly related to the incorporation of the phosphorylated deoxyribonucleoside derivative into viral cDNA during reverse transcription (39, 67). We have included this analogue as a negative control for antiviral mutagenesis in our RNA viral targets, as it is not predicted to be a substrate of RNA polymerases. We first investigated the toxicities of these compounds on Vero cells (Fig. 1). Only prolonged treatments with 5-fluorouracil (48 h) led to cell death rates above 20%. The remaining drugs only showed modest or no effect on cellular viability even after prolonged exposures to the highest concentration tested (800 μM).

**FIG 2:**
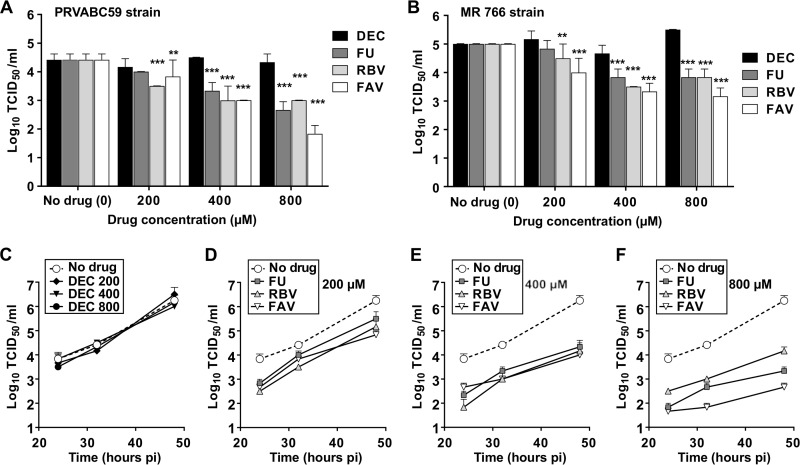
Favipiravir, ribavirin, and 5-fluorouracil inhibit ZIKV replication. (A and B) ZIKV titers obtained after infection of confluent Vero cell monolayers in the absence (drug concentration of 0 in the abscissa) or presence of each drug at the concentrations indicated. Cells were infected at an MOI of 0.01 and the supernatants collected at 32 h postinfection for titration. (A) ZIKV of Asian lineage (strain PRVABC59); (B) ZIKV African lineage (strain MR 766). Statistically significant differences are highlighted with asterisks (**, *P* < 0.01; ***, *P* < 0.001; 2-way ANOVA). Every value represents the average of the results from at least three biological replicas (± standard error of the mean [SEM]). Decitabine (DEC) values are shown as black bars, 5-fluorouracil (FU) as dark gray, ribavirin (RBV) as light gray, and favipiravir (FAV) as white bars. (C) Replication kinetics of ZIKV (Asian lineage, strain PRVABC59) in the presence of different concentrations of decitabine (DEC). Every value represents the average from virus titer determinations of at least three independent biological replicas (± SEM). Each symbol illustrates a different concentration of decitabine used in the assay, as follows: diamond, 200 μM; inverted triangle, 400 μM; black circle, 800 μM. (D to F) Replication kinetics of ZIKV (Asian lineage) in the presence of FU (dark-gray squares), RBV (light-gray triangles), and FAV (white inverted triangles) are compared to those in untreated infected cultures (white circles, dashed lines). Every value is obtained from the analysis of at least three biological replicas (± SEM). Each panel depicts viral replication kinetics in the presence of inhibitors at different concentrations, 200 μM (D), 400 μM (E), or 800 μM (F).

Treatment of infected cells with all the ribonucleosides, i.e., favipiravir, ribavirin, and 5-fluorouracil, led to significant inhibition of ZIKV replication (Fig. 2). These molecules exhibited similar antiviral activities when using an epidemic strain isolated in the Americas (Asian lineage; Fig. 2A) and an African isolate (Fig. 2B). As predicted, decitabine showed no effect on ZIKV replication, further supporting the idea that the antiviral activity elicited by the ribonucleosides is directly related to their incorporation by the viral RNA polymerase (Fig. 2C). Further analysis of ZIKV replication kinetics in the presence of different concentrations of each drug suggests that these molecules exhibit a similar inhibitory capacity. However, favipiravir seems to elicit a stronger inhibitory activity than ribavirin and 5-fluorouracil when higher concentrations are used (Fig. 2D to F).

### Favipiravir, ribavirin, and 5-fluorouracil cause effective ZIKV extinction after serial passage in Vero cells.

**FIG 3:**
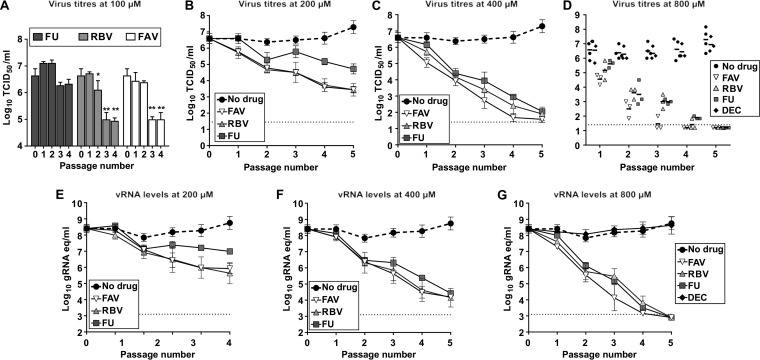
Favipiravir, ribavirin, and 5-fluorouracil cause efficient extinction of ZIKV during serial passages in cell culture. ZIKV was serially passaged in the absence (black circles, dashed lines), or in the presence of mutagenic drugs at 100 μM (A), 200 μM (B and E), 400 μM (C and F), or 800 μM (D and G). Three independent lineages of passages were performed for each drug and concentration tested. Serial passages were carried out with 100 μl of the cell culture supernatant recovered from the previous infection passage (corresponding to 1/10 of the total volume collected). The different graphs show the virus titers determined by TCID_50_ assay (A to D) and the genome copy equivalents obtained by quantitative PCR (qPCR) assays (E to G) that were found along serial passages of ZIKV. A black diamond represents ZIKV titers found in the supernatants of cultures treated with decitabine (DEC); dark-gray squares illustrate 5-fluorouracil (FU)-treated series; light gray triangles, ribavirin (RBV); and white inverted triangles, favipiravir (FAV). Every value represents the average of virus titrations or viral genome copy equivalents (gRNA eq) from at least three biological replicas obtained from independent series of passages (± SEM). In panel D, individual values obtained from each lineage are represented to better illustrate independent events of virus extinction.

**FIG 4:**
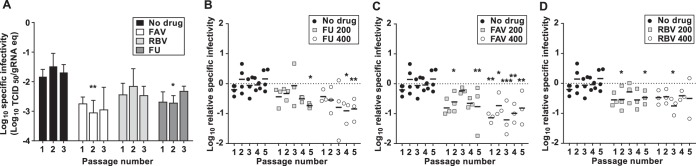
ZIKV populations passaged in cell cultures treated with mutagenic compounds exhibit decreased specific infectivity. Specific infectivity values were calculated as the ratio of infectious virus units (TCID_50_) to viral genome copies (quantified by qPCR) in every biological sample. (A) Values found in untreated populations (black bars) or populations treated with FU (dark-gray bars), RBV (light gray), or FAV (white) during three passages in the presence of each drug at 800 μM. The values are the averages of the results from three independent biological replicas (± SEM). (B to D) The values found during 5 consecutive passages in untreated populations (black circles) or populations treated with each mutagen at different concentrations (200 μM [light-gray squares] or 400 μM [white circles]) are represented. Each graph illustrates independent values obtained in three independent lineages for each drug and concentration tested, 5-fluorouracil (B), ribavirin (C), and favipiravir (D). Statistically significant differences are represented (*, *P* < 0.05; **, *P* < 0.01; ***, *P* < 0.001; 2-way ANOVA).

We further analyzed the antiviral efficacies of these drugs against ZIKV during prolonged treatment by testing their capacity to abolish infectivity during consecutive viral passages in cell culture. Sequential infections of ZIKV in the presence of these drugs resulted in a total loss of infectivity when a final concentration of 800 μM was used (except for decitabine; Fig. 3). The complete extinction of ZIKV infectivity was replicated in three independent lineages of passages in each drug (Fig. 3D and G). Favipiravir eliminated ZIKV in a faster manner (undetectable viral levels reported after 4 passages for all the three independent lineages) than ribavirin and 5-fluorouracil (all three lineages were extinct after 5 passages). The extinction of ZIKV populations in these samples was confirmed by performing an additional blind passage in cell culture in the absence of mutagens (data not shown). We did not observe any detectable infectivity or viral RNA in the samples recovered, confirming that these three nucleosides completely eliminate ZIKV infectivity. Serial passage of ZIKV in cells treated with ribavirin or favipiravir at lower concentrations (100 to 400 μM) also resulted in a gradual decrease in infectivity, with the two drugs presenting similar efficacies (Fig. 3A to C, E, and F). Unlike favipiravir and ribavirin, ZIKV exhibited lower susceptibility to passages in the presence of 5-fluorouracil, suggesting that this molecule is a weaker inhibitor or mutagen for this virus. As anticipated, decitabine had no effect on infectivity, as the viral titers measured during serial transfers were similar to those found in passages of virus in the absence of drug (Fig. 3D and G). A typical signature of lethal mutagenesis is that viral populations isolated in passages preceding extinction show reduced specific infectivity (30, 68). To investigate whether ZIKV populations treated with these compounds manifested any alteration in their specific infectivity values, we determined the proportion of infectious particles relative to the total number of viral RNA molecules in the sample. We confirmed that viral populations rescued after treatment with all three drugs exhibited lower specific infectivity than the untreated viruses, and most significantly those treated with favipiravir (Fig. 4).

### USUV shows a different sensitivity pattern to nucleoside analogues from that with ZIKV.

**FIG 5:**
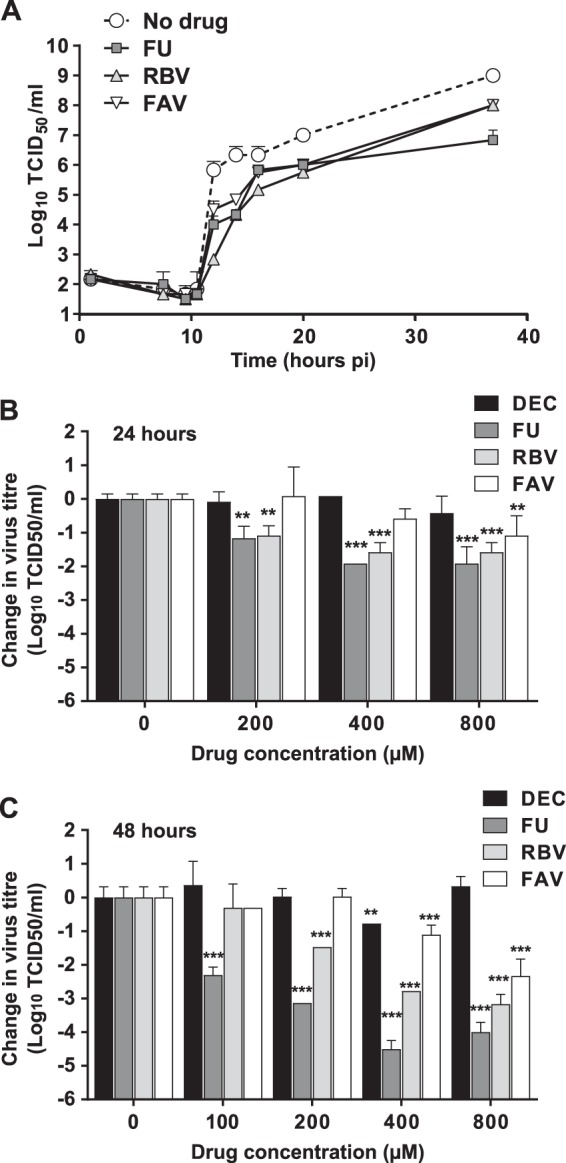
Mutagenic nucleosides inhibit USUV replication in Vero cells. (A) Single-cycle replication kinetics of USUV treated with FU (dark-gray squares), RBV (light-gray triangles), or FAV (white inverted triangles) at 800 μM each, compared to that of untreated virus. Vero cells were inoculated at an MOI of 5 TCID_50_ per cell. Cellular supernatants were collected at different time points after infection. Every value in the graph is the average of the results from at least three biological replicas (± SEM). (B and C) USUV titers obtained after multiple rounds of virus replication in Vero cells in the absence (0) or presence of increasing concentrations of each drug. To ensure that the virus titers are the result of several rounds of replication, we employed a low MOI to infect the cells (0.1 or 0.01). Statistically significant differences are represented (**, *P* < 0.01; ***, *P* < 0.001; 2-way ANOVA). Each value in the graph is calculated as the average virus titer obtained from at least three independent biological replicates (± SEM). Virus titers obtained in infected cells treated with DEC are represented as black bars, titers in FU-treated cells are in dark gray, RBV cells are in light gray, and FAV cells are in white. (B) Vero cell monolayers were infected at an MOI of 0.1 and supernatants collected for titration at 24 h postinfection. (C) Supernatants of infected cells were collected for virus titer analysis at 48 h postinfection. To ensure that virus titers were obtained during the exponential-growth phase, we used an MOI of 0.01 instead of 0.1.

To elucidate whether these drugs are also broadly effective against other flaviviruses, we analyzed their antiviral activity on Vero cells infected with USUV. All three nucleosides (favipiravir, ribavirin, and 5-fluorouracil) that inhibited ZIKV replication also manifested antiviral activity on USUV (Fig. 5). Likewise, treatment with decitabine exhibited no effect on USUV replication (Fig. 5B and C). In contrast to what we had observed with ZIKV, 5-fluorouracil was the most effective compound against USUV (Fig. 5). Single-cycle infection kinetics experiments with drugs at 800 μM revealed that 5-fluorouracil antiviral activity becomes more prominent at later replication time points (Fig. 5A). This is also observed when the same drugs are tested against USUV during multiple cycles of infection (infections at low multiplicity of infection [MOI]). The relative activity of 5-fluorouracil compared to those of ribavirin and favipiravir is substantially larger at 48 h than at 24 h (Fig. 5B and C). This observation may be in agreement with a larger accumulation of mutations during larger number of replication cycles taking place during 48 h.

**FIG 6:**
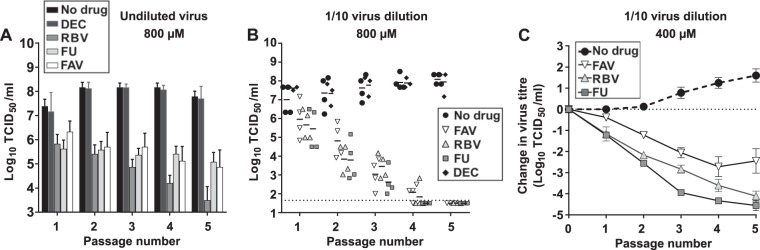
Extinction of USUV by nucleoside drugs requires viral sample dilution during serial transfers. (A) Passage of USUV in cells cultured in the presence of mutagenic drugs at 800 μM. In each passage, 100 μl of neat sample collected from the previous infection was applied to a new monolayer of cells. The bars are the average of titers obtained from four independent series. The values show the evolution of infectivity in cells treated with FU (gray), RBV (light gray), FAV (white), and DEC (dark gray), as well as in untreated cells (black). (B) FAV, RBV, and FU can lead USUV to extinction during serial passage of diluted viral samples. In each passage, 100 μl of a 10-fold diluted sample collected from the previous passage was applied to the following infection round. Different symbols in the graph show the evolution of infectivity in cells treated with FU (dark-gray squares), RBV (light-gray triangles), FAV (white inverted triangle), and DEC (black diamond) at a concentration of 800 μM. Virus titers in untreated USUV cultures are also represented (black circle). Individual values obtained from four independent lineages are represented to better illustrate independent events of virus extinction (two lineages for DEC). (C) Same as in panel B, but a concentration of 400 μM was used for each drug treatment on three independent lineages (DEC was not tested at this concentration).

Serial passage of USUV in the presence of each ribonucleoside drug led to sustained decreases in virus titers but not to complete viral extinction (Fig. 6A). Virus lineages treated with ribonucleosides showed significantly lower titers (3 to 4 log_10_ on average) than during passages in decitabine or in the absence of drug. The lowest values were observed in the presence of 5-fluorouracil, further suggesting that this compound is most effective against USUV during serial passage in cell culture (Fig. 6A).

The apparent lower susceptibility to nucleoside analogues in USUV than in ZIKV could be related to generally larger virus yields in infections with USUV. Previous studies on unrelated foot-and-mouth disease virus (FMDV) suggested that the efficacy of lethal mutagenesis can be affected by the viral load; hence, extinction will be favored when the infection is initiated with a lower viral dose (33). Thus, we decided to investigate whether passages at a lower infectious dose may also facilitate USUV extinction. We found that all three ribonucleoside analogues (800 μM) can reproducibly drive USUV to extinction in four independent replicas when a 10-fold dilution was applied before each transfer (Fig. 6B). Viral sample dilution during sequential passages in the absence of drugs or in the presence of decitabine did not affect infectivity (virus titers in the range of 10^6^ to 10^8^ 50% tissue culture infective dose [TCID_50_] per ml; Fig. 6B). When drugs were used at a lower concentration (400 μM), the complete elimination of USUV was only observed in some sporadic cases. Both ribavirin and 5-fluorouracil caused USUV extinction in two out of three lineages, while favipiravir never led to the complete loss of infectivity in any of three series analyzed (not shown). Further analysis revealed that treatment with 5-fluorouracil resulted in significantly larger decreases in virus titers than passages in the presence of other drugs at 400 μM (Fig. 5C; at passage 3, *P* < 0.01 for ribavirin versus 5-fluorouracil, and *P* < 0.001 for ribavirin versus favipiravir, 2-way analysis of variance [ANOVA]).

### Differences in sensitivities to nucleoside analogues in USUV and ZIKV are associated with alterations in their mutational patterns.

**FIG 7:**
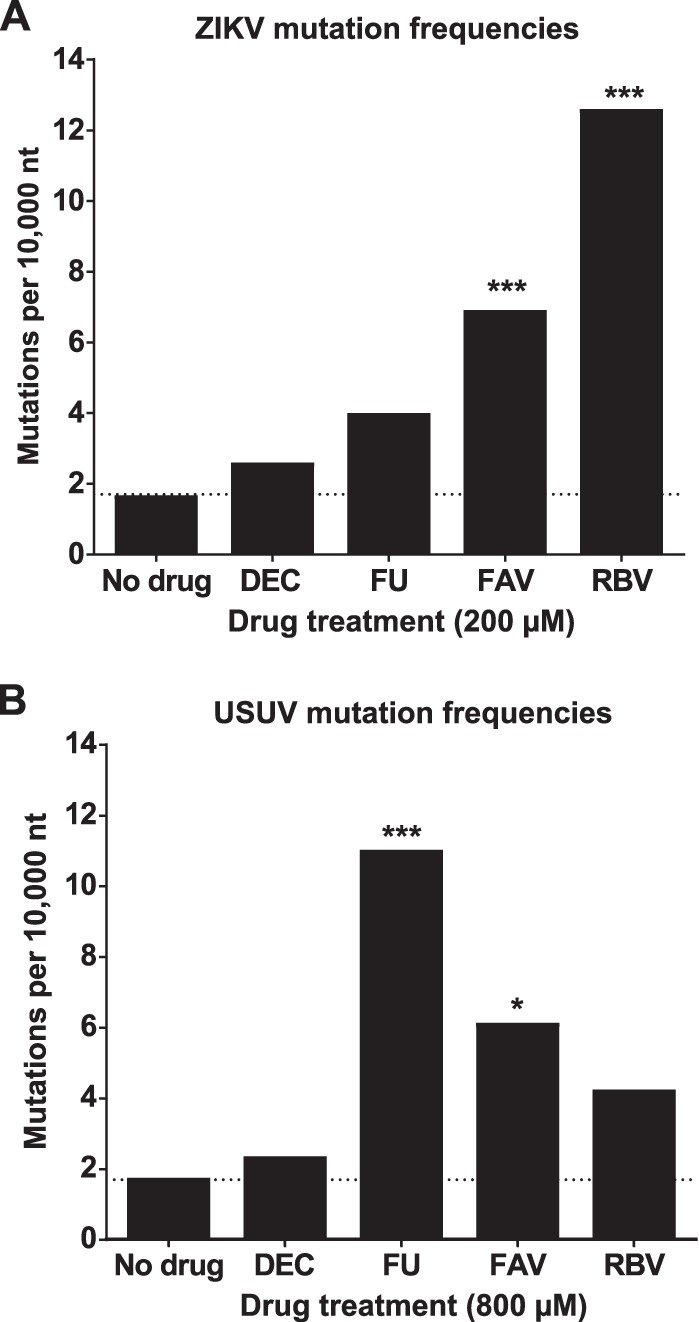
Treatment with mutagenic nucleosides leads to an increase in the mutation frequencies of ZIKV and USUV populations. Mutation frequencies found in ZIKV (A) and USUV (B) populations are represented as the average number of nucleotide substitutions identified every 10,000 nucleotides analyzed. (A) To analyze the ZIKV mutation profile, we isolated individual sequences from samples recovered after five passages in the presence of 200 μM 5-fluorouracil (FU), favipiravir (FPV), or ribavirin (RBV), 800 μM decitabine (DEC), or in the absence of any drug. Total viral RNA was extracted and RT-PCR amplified, and the individual cDNA sequences were isolated by cloning in the pCR-Blunt cloning vector following procedures described in Materials and Methods. A total of 29,818, 23,120, 22,497, 27,476, and 20,558 nucleotides (nt) for populations recovered after passage in the presence of no drug, DEC, FU, FAV, and RBV, respectively, were sequenced. (B) Mutation frequencies in USUV populations isolated after 5 passages in the presence of each drug at 800 μM. The mutation frequency values are based in the analysis of a total of 45,337, 33,947, 34,453, 34,218, and 25,897 nucleotides in populations collected after passage in the presence of no drug, DEC, FU, FAV, and RBV, respectively. Statistically significant increases compared to untreated populations are indicated (*, *P* < 0.05; ***, *P* < 0.001; Mann-Whitney U test).

To investigate whether the antiviral activities observed during treatment with favipiravir, ribavirin, and 5-fluorouracil are connected to their predicted mutagenic activity, we analyzed the mutation frequencies of both ZIKV and USUV rescued after 5 passages in the presence of these compounds. Since treatment at high concentrations (400 and 800 μM) resulted in a rapid loss of ZIKV infectivity, we isolated and analyzed viral RNA from the supernatant of infected cells after 5 consecutive passages during exposure to drugs at 200 μM (Fig. 3 and 7). Both favipiravir- and ribavirin-treated virus populations displayed significantly higher mutation frequencies (*P* < 0.001; Mann-Whitney test for the distribution of mutations per clone) than those untreated or isolated after treatment with 5-fluorouracil or decitabine. These results are in agreement with the relative antiviral efficacy of each nucleoside drug (Fig. 3 and 7A). The highest mutation frequencies were observed in the ribavirin- and favipiravir-treated ZIKV population (7- and 4-fold higher than in untreated virus, respectively). This positively links antiviral activity with mutagenesis, as the largest decreases in ZIKV titers observed during serial passages in the presence of each drug (at 200 μM) were observed with ribavirin and favipiravir (Fig. 3B and E and 7A). In the 5-fluorouracil-treated population, we only found a modest (2-fold) increase in the mutation frequency (Fig. 7A), reflecting the milder antiviral behavior of this compound. Treatment with decitabine did not significantly alter the mutation frequency (Fig. 7A), further suggesting that this compound is not affecting RNA replication.

Similarly, we found that the largest mutation frequencies in USUV were observed in populations collected after serial passage in the presence of 5-fluorouracil (Fig. 6. and 7B). Viral populations recovered after 5 passages in the presence of 5-fluorouracil (800 μM) showed 6-fold larger mutation frequencies than USUV passaged in the absence of drug. Ribavirin and favipiravir led to modest increases in the mutational loads, also in agreement with their milder antiviral activities against USUV (2 and 3-fold, respectively; Fig. 6 and 7B).

Further analysis revealed the expected transition biases typically found in viruses treated with the same drugs (25, 30, 33, 35, 69–72). Thus, we observed slightly higher rates of G-to-A and C-to-U transitions in viruses treated with favipiravir, while the opposite changes (A-to-G and U-to-C) were identified in 5-fluorouracil-treated viruses (Tables 1 and 2). The only exception to these typical transition mutational patterns was obtained in ZIKV populations treated with ribavirin (Table 1), with higher frequencies in A-to-G and U-to-C nucleotide substitutions (when the [A-to-G + U-to-C]/[G-to-A + C-to-U] ratio is 2). In contrast, USUV treated with ribavirin exhibited the expected mutational bias, with larger numbers of G-to-A and C-to-U transitions (Table 2).

**TABLE 1:** Mutation types found in ZIKV populations treated with different drugs

Drug^*a*^	No. of mutations in each population	No. of nucleotides sequenced in each population	No. of mutations by type^*b*^					Transition frequency (10 × ^−4^)^*c*^			
			A to G	U to C	G to A	C to U	Tv	A to G	U to C	G to A	C to U
No drug	5	29,818	2	0	1	0	2	**2.7**	<1.5	1.2	<1.4
DEC	6	23,120	2	1	2	1	0	**3.4**	1.9	3.1	1.7
FAV	18	27,476	1	5	5	4	4	1.5	**8.2**	6.6	5.9
RBV	26	20,558	8	6	2	5	5	**15.4**	13.1	3.5	9.8
FU	9	22,497	4	1	2	1	1	**7.0**	2.0	3.2	1.8

a ZIKV populations were serially passaged five times in the absence of drug (no drug) or in the presence of either decitabine (DEC), favipiravir (FAV), ribavirin (RBV), or 5-fluorouracil (FU) at a concentration of 200 μM.

b Different type of mutations found in the analysis. Given are the number of times that each transition type is found in the analysis. Tv indicates the number of transversions found in the analysis.

c Transition frequencies found in populations treated with mutagenic drugs. These numbers have been normalized to the nucleotide composition in the sequenced amplicon. Highlighted in bold is the most frequent transition in each sample.

**TABLE 2:** Mutation types found in USUV populations treated with different drugs

Drug^*a*^	No. of mutations in each population	No. of nucleotides sequenced in each population	No. of mutations by type^*b*^					Transition frequency (10 × ^−4^)^*c*^			
			A to G	U to C	G to A	C to U	Tv	A to G	U to C	G to A	C to U
No drug	8	45,337	2	1	2	0	3	**1.8**	1.0	1.6	<0.9
DEC	8	33,947	1	2	0	2	3	1.2	**2.6**	<1.1	2.4
FAV	20	34,218	3	5	5	6	1	3.5	6.4	5.2	**7.2**
RBV	11	25,897	0	2	4	4	1	<1.5	3.4	5.5	**6.4**
FU	37	34,453	10	15	1	3	8	11.5	**19.2**	1.0	3.6

a USUV populations were serially passaged five times in the absence of drug (no drug) or in the presence of either decitabine (DEC), favipiravir (FAV), ribavirin (RBV), or 5-fluorouracil (FU) at a concentration of 800 μM.

b Different type of mutations found in the analysis. Given are the number of times that each transition type is found in the analysis. Tv indicates the number of transversions found in the analysis.

c Transition frequencies found in populations treated with mutagenic drugs. These numbers have been normalized to the nucleotide composition in the sequenced amplicon. Highlighted in bold is the most frequent transition in each sample.

## DISCUSSION

Lethal mutagenesis has been posited as an alternative strategy to the current therapies based on classical antiviral drugs. Several lines of evidence sustain that the antiviral properties of ribavirin and favipiravir *in vivo* can be, at least in part, connected to their mutagenic activity (25, 26, 50). These data encourage further study on the development of antiviral compounds with reduced toxicity, improved pharmacokinetics, and higher specificity for the viral polymerases (25, 26, 50). In this study, we have investigated the sensitivity to mutagenesis of two flaviviruses that have recently emerged as serious threats to public health, ZIKV and USUV. Both pathogens were highly sensitive to the exposure of three mutagenic nucleosides, i.e., 5-fluorouracil, favipiravir, and ribavirin, although they exhibited different degrees of susceptibility to them. ZIKV was inhibited more efficiently by ribavirin and favipiravir, while USUV replication was affected to a greater extent by 5-fluorouracil. These inhibition profiles correlate with the relative increase in the mutation frequencies observed in populations rescued after treatment, supporting the idea that their antiviral efficacy is closely associated with their mutagenic activity.

A possible explanation for the different sensitivities of USUV and ZIKV to mutagenic drugs is that the molecular determinants that regulate nucleotide recognition and discrimination in their respective polymerases vary. As for other RNA viruses, flavivirus genome replication is an error-prone process catalyzed by the viral polymerase contained in the nonstructural protein 5 (NS5) (64, 73–77). Even though further analysis is needed to confirm this possibility, our data suggest that ZIKV is more prone to misincorporate purine analogues, such as ribavirin and favipiravir, while USUV shows a preference for pyrimidine substrates, like 5-fluorouracil. If certain, this information could be instrumental in the rational screening of nucleoside analogues to effectively control each flavivirus. Many recent studies have contributed to the better understanding of the molecular biology of ZIKV replication, including the structural and biochemical characterization of the viral polymerase NS5 (74–78). However, there is limited knowledge on USUV replication, with no molecular data on its viral polymerase. Future studies are thus needed to elucidate the molecular determinants responsible for the different sensitivities of these flaviviruses to mutagenic nucleosides.

Additional evidence hinting at molecular differences in ZIKV and USUV polymerase fidelity can be inferred from the mutation patterns found in viral populations after treatment with ribavirin (Tables 1 and 2). We observed the typical dominance of G-to-A and C-to-U transitions in USUV and an unexpected prevalence of the opposite transition types in ZIKV (the [G-to-A + C-to-U]/[A-to-G + U-to-C] ratios were 4 and 0.5 for USUV and ZIKV, respectively). It has been suggested that the larger proportion of G-to-A and C-to-U transitions in viruses treated with ribavirin (included USUV) is possibly connected to the additive effect of mutagenesis by incorporation of ribavirin-triphosphate into viral RNA and depleted intracellular GTP pools as a consequence of inosine-5′-monophosphate dehydrogenase (IMPDH) inhibition by ribavirin (69, 79, 80). Although ribavirin-triphosphate is efficiently incorporated during viral RNA synthesis opposite to U and C, in such a low intracellular GTP concentration scenario, it may be preferentially incorporated opposite to C, hence leading to those biases (79). The dominance of A-to-G and U-to-C mutations in ribavirin-treated ZIKV populations suggests that its polymerase may be favoring a different base pairing behavior of ribavirin than in other viruses with independence on intracellular nucleotide pools. Other plausible scenarios may include differences in host cell rearrangements that indirectly affect virus mutability by the same drugs (e.g., alterations in the expression of cellular proteins associated with nucleotide uptake and its metabolism). Further investigations are needed to clarify the mechanism underlying these differences.

We deem that this study can stimulate an additional investigation on the therapeutic value of mutagenic drugs against flaviviruses and, in particular, for the treatment of persistent flaviviral disease (8, 81–83). Mutagenic drugs seem to be especially effective against persistent infections, with major evidences of lethal mutagenesis obtained during treatment of chronically infected hosts, i.e., HCV-infected patients treated with ribavirin, and mice persistently infected with norovirus and treated with favipiravir (25, 50). A tentative explanation for the better efficacy of mutagenic drugs in this context may be linked to potentially larger accumulation of mutations during longer periods of exposure to drugs (68, 84). A vast range of flavivirus-associated disorders have been connected with persistent infection, including ZIKV (8, 81–83, 85). ZIKV infects a broad range of cell types and tissues, including the reproductive tract and the central nervous system (8, 82, 85). Persistent replication in these tissues has been connected to diverse severe conditions, such as end-organ disease, platelet-related illness, and potentially blinding uveitis (8). Persistent ZIKV in the reproductive tissue is possibly responsible for a growing number of cases of sexually transmitted disease, with some studies predicting that this may become a major route of infection in the future (10, 86–92). Vaginal mucosa infection has also been related to fetal infection, further highlighting the impact of ZIKV persistence in the genital tract (9, 93). It is therefore tempting to investigate the potential therapeutic value of lethal mutagenesis in the treatment of persistent infection in the reproductive tract mucosa.

It remains unclear whether ribavirin and favipiravir will elicit efficient antiviral mutagenesis in the central nervous system (CNS), with recent data showing different efficacies of these drugs in neuron-derived cell culture systems. While some studies demonstrate that ZIKV replication remains unaffected by these drugs in stem cell-derived neurons, other investigations have proven their inhibitory activity during the treatment of neural progenitor cell lines (59, 60). The different efficacies of these molecules could be linked to possible divergences in the cellular uptake or metabolism of nucleoside drugs in these cell lines. Thus, for the control of neurotropic disease, it would be desirable to identify mutagenic compounds with improved pharmacological properties in the CNS. Alternatively, a combination of mutagenic drugs that are effective in controlling the infection outside the CNS (favipiravir, ribavirin, and 5-fluorouracil), together with effective inhibitors in the neuronal tissue (60, 94–96), could lead to improved approaches to control ZIKV hidden in different body compartments.

Our study also encourages the investigation of 5-fluorouracil as a therapeutic drug for the control of USUV infection in an eventual epidemic spillover to humans. Although severe disease in humans is rare, there is growing evidence that cases of neurologic disorders associated with USUV have been historically misdiagnosed as WNV (22, 97). The diagnosis of disease caused by USUV is further complicated by the resemblance of pathology to WNV cases and the serological cross-reactivity in diagnostic tests (11, 13, 18, 22–24, 97, 98). A mouse model for USUV infection has been recently established, and it represents a valuable tool to test the *in vivo* antiviral effect of 5-fluorouracil or novel antivirals (98, 99). These studies can be extended to the potential treatment of severe disease caused by closely related flaviviruses, such as WNV and Japanese encephalitis virus, for which antiviral therapies are not available.

## MATERIALS AND METHODS

### Cells, viruses, and protocols for infections.

We used two different ZIKV strains purchased from the American Type Cell Culture (ATCC). The majority of the experiments described in this paper were performed with an isolate from the Asian lineage, collected during the recent epidemics in the Americas (strain PRVABC59, Puerto Rico, 2015, ATCC reference number VR-1843). In addition, for some experiments indicated here, we used the first ZIKV strain ever isolated as our reference African lineage virus (Uganda, 1947, strain MR 766, ATCC reference number VR-1838). The USUV strain (939/01) used in this study was initially isolated from infected birds in Austria in 2001 and was kindly provided by Giovanni Savini, Istituto G. Caporale, Italy (100).

We used African green monkey kidney epithelial cells (kindly provided by Sylvie Lecollinet, ANSES, France), namely, Vero cells, for ZIKV and USUV propagation, titration, and viral infections in the presence or absence of mutagenic compounds. Viral infections were performed as follows: on the day preceding virus inoculation, we seeded 24-well plates with 4 × 10^5^ cells per well in the presence of 1 ml of complete medium containing 5% (vol/vol) fetal bovine serum (FBS; Sigma), 100 units/ml penicillin-streptomycin (Thermo Fisher), and 1 mM HEPES in high-glucose Dulbecco's modified Eagle medium (DMEM; Thermo Fisher). Cells were incubated overnight at 37°C with a 5% CO_2_ concentration. On the following day, the supernatant was removed from each plate and replaced with 250 μl of fresh medium containing 1% FBS. Then, 100 μl of virus sample (ZIKV or USUV) was applied to the cell monolayer at the multiplicity of infection (MOI) indicated, and virus adsorption was allowed for 1 h at 37°C with 5% CO_2_. The inoculum was removed and the cells washed with complete medium to eliminate unattached virus. Cells were then covered in 1 ml of medium containing 1% FBS, and supernatants from infected cultures collected at different time points.

### Virus titration.

Virus samples were titrated by 50% tissue culture infectious dose (TCID_50_) assays. To this aim, 10^4^ Vero cells in 100 μl were seeded in 96-well plates in the presence of medium containing 1% FBS. On the following day, 100 μl of 10-fold serial dilutions of each sample was applied to each well, reaching a final volume of 200 μl. The virus titers were determined by scoring the number of infected wells showing apparent cytopathic effect at 4 to 5 days postinfection, and using the Spearman & Kärber algorithm (101).

### Cell culture infections in the presence of antiviral compounds.

For infections in the presence of drugs, cell culture supernatants were removed, and 250 μl of 1% FBS complete medium containing 100 to 800 μM decitabine (5-aza-2′-deoxycytidine; Selleckchem), 5-fluorouracil (2,4-dihydroxy-5-fluoropyrimidine; Sigma-Aldrich), ribavirin [1-(β-d-ribofuranosyl)-1H-1,2,4-triazole-3-carboxamide; Sigma-Aldrich], or favipiravir (6-fluoro-3-hydroxy-2-pyrazinecarboxamide; Atomax) was added to each well. Cells were then inoculated with 100 μl of virus at the MOI indicated for each experiment in the corresponding section and incubated for 1 h at 37°C and 5% CO_2_. Supernatants were removed, cells were washed to eliminate unattached virus, and 1 ml of fresh medium (1% FBS) containing each drug at the desired concentration was added. Cell culture supernatants were collected at 24 to 48 h postinfection for subsequent analyses.

For experiments involving serial passage of viruses in the presence of nucleoside analogues, the first infection with ZIKV or USUV was carried out at an MOI of 0.1 TCID_50_/cell. In sequential passages, 100 μl of neat virus from the supernatant of the previous passage (which represents 1/10 of the total volume collected) was applied to a new monolayer of cells. For experiments involving transfers of diluted USUV, we used in each transfer 100 μl of a preparation containing a 10-fold dilution of the viral sample collected in the previous passage, as described in previous work (25).

### Viral RNA extraction, reverse transcription-PCR amplification, and mutation frequency analysis.

Viral RNA was extracted from 100 μl of viral sample supernatants using the GeneJET RNA purification kit (Thermo Fisher). For the calculation of mutation frequency values, we followed previously described protocols (25, 102, 103). Briefly, 4 μl of purified RNA was reverse transcribed in 20 μl final volume using SuperScript III (Roche), as indicated by the manufacturer. Three microliters of cDNA was then PCR amplified using high-fidelity KOD polymerase (Toyobo). To this aim, we used primers spanning genomic positions 2976 to 3009 and 5052 to 5074 in USUV and 6465 to 6486 and 7646 to 7677 in ZIKV. PCR products were gel purified using the PureLink Quick gel extraction kit (Invitrogen) and then ligated into plasmid PCR Blunt using the Zero Blunt PCR cloning kit (Thermo Fisher). Positive E. coli colonies were identified by PCR screening with primers flanking the vector-cloning site and DreamTaq DNA polymerase (Thermo Fisher). The resulting PCR products, corresponding to individual ZIKV or USUV cDNA clones, were Sanger sequenced and the mutation frequency in each population calculated.

### Quantitative PCR analysis of virus populations.

The number of ZIKV genomic RNA molecules in different biological samples was quantified using primers and a FAM-TAMRA (6-carboxyfluorescein–6-carboxytetramethylrhodamine) probe targeting the ZIKV E gene (positions 1214 to 1244), following protocols previously described (93). For the amplification protocol, we used TaqMan Fast Virus 1-step master mix (Thermo Fisher) and one-step reaction reverse transcription-PCR (RT-PCR) amplification conditions, with a reverse transcription step (30 min at 48°C), followed by 1 min of incubation at 95°C and 40 amplification cycles of 15s at 95°C and 1 min at 60°C. For the quantification of USUV RNA, we used a FAM-TAMRA probe targeting the NS5 gene (positions 9297 to 9318) and primers previously described (104). The same RT-PCR cycle amplification protocol described above for the detection of ZIKV was used for USUV.

### Statistical analysis.

Statistical significance was assessed using GraphPad Prism 7, as specified in the figure legends. For the statistical analysis of mutation frequencies, we employed a Mann-Whitney test that compares the ranked scores of the number of mutations found in individual clones grouped by population, as previously described (105).
